# Anterior Esthetic Rehabilitation with Computer-Aided Design/Computer-Aided Manufacturing Zirconia: A Case Report

**DOI:** 10.7759/cureus.59936

**Published:** 2024-05-08

**Authors:** Prasanna R Sonar, Aarati Panchbhai, Ankita Pathak

**Affiliations:** 1 Oral Medicine and Radiology, Sharad Pawar Dental College and Hospital, Datta Meghe Institute of Higher Education & Research, Wardha, IND; 2 Prosthodontics, Sharad Pawar Dental College and Hospital, Datta Meghe Institute of Higher Education & Research, Wardha, IND

**Keywords:** anterior esthetics, zirconia, cad cam, esthetics, digital dentistry

## Abstract

In layered zirconia prosthesis, the choice of zirconia composition, framework design, and shade is closely related to the properties of the abutments. This interdependence emphasizes how crucial it is to take a deliberate and tailored approach to meet the unique needs of every therapeutic circumstance. To successfully treat anterior teeth and achieve restorations that look natural, challenges such as incorrect size and shape, atypical gingival contour, and unsightly hues need to be resolved. Ceramodetal restorations have occasionally allowed for the most appealing, authentic replication of natural teeth, despite its proven strength, endurance, and improved aesthetics. Due to their superior cosmetic results, metal-free materials have been used for anterior rehabilitation. Materials like dental zirconia, which offers excellent aesthetics and desired mechanical characteristics, have emerged in response to the increased need for visually appealing and metal-free alternatives. In this case study context, experiences in the clinic using multilayer zirconia prostheses intended exclusively for anterior teeth are discussed.

## Introduction

A pleasant smile and face are essential for improving one's self-esteem and general well-being [1,2]. One of the main objectives of restorative care is aesthetics. The position of the teeth, color, shape, and gingival tissue, as well as the appropriate placement of the lips, all affect how attractive a smile is [3-6]. Therefore, when prosthetic rehabilitation is required, an integrated assessment of all components should be carried out [4,5]. The restoration of maxillary anterior teeth requires a thorough grasp of the characteristics of the natural teeth and a meticulous plan for rehab. This consists of a radiological and clinical evaluation, study models with diagnostic waxing, and collaboration between an interdisciplinary team and aesthetic rehabilitation [7-10]. When creating a prosthesis that closely resembles the natural dentition, the right materials and processes must be chosen to achieve the best potential aesthetic outcome.

Zirconia is one of the dental ceramics whose use in dentistry has increased significantly due to its white shade and practical results. Developments in ceramic systems enable long-lasting and aesthetically pleasing prostheses. The zirconia ceramic technique allows for permanent prostheses with exceptional aesthetic results [11]. These are a great substitute for metal-structured fixed prostheses. A noticeable trend toward monolithic zirconia crowns has resulted from problems with breakage, fracture, delamination, and chipping that plague conventional fixed prosthesis, which frequently use a zirconia substructure with veneering porcelain. Due to its many benefits, monolithic zirconia crowns, which are well-known for their entire contour design, are becoming more and more common. When it comes to maxillary anterior teeth, where obtaining the best possible aesthetics and functionality is crucial, it is especially important because monolithic zirconia crowns are preferred [12-15].

In modern dentistry, zirconia restorations can only be created using computer-aided design/computer-aided manufacturing (CAD/CAM). The case study also discusses the utilization of CAD/CAM zirconia material in treatment, paying particular attention to a patient's needs. This case study provides a thorough overview of the effective use of zirconia all-ceramic crowns in the treatment of a female patient. The final prosthesis not only satisfies exacting aesthetic requirements but also guarantees complete functional competency, improving the patient's mental and psychological state. When designing ceramic crowns, the dentist must be considerate of the patient's aesthetic preferences because individual symmetry and aesthetic preferences may not be satisfied by technical precision alone. Successful outcomes necessitate careful consideration of anatomical factors in addition to a deep comprehension of the patient's desires.

## Case presentation

**Figure 1:**
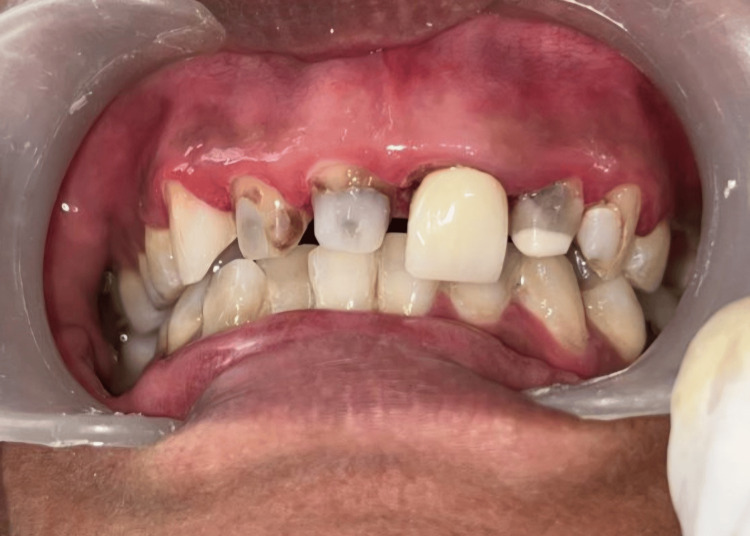
Frontal view. Image credit: Prasanna Sonar

**Figure 2:**
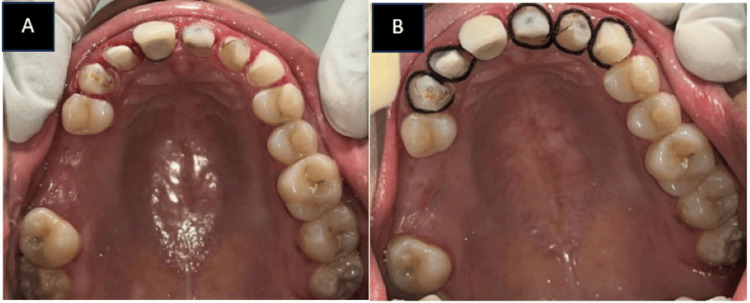
A) Tooth preparation with 11 12 13 22 23; B) gingival retraction with retraction cord. Image credit: Prasanna Sonar

**Figure 3:**
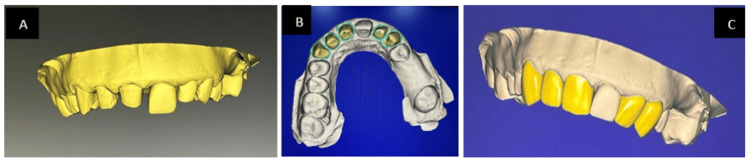
A) Scanning. B) Locating the margins. C) Designing. Image credit: Prasanna Sonar

**Figure 4:**
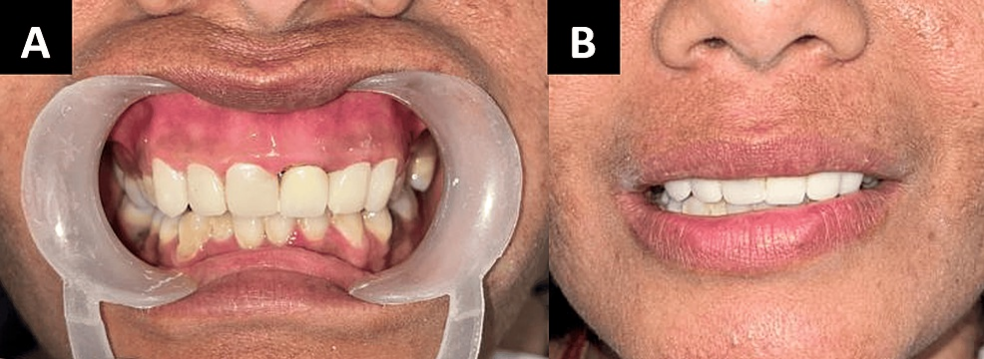
A) Post-treatment photograph. B) Post-treatment photograph. Image credit: Prasanna Sonar

Figure 1 in the case report depicts a 31-year-old female patient who requires many fillings in her maxillary anterior teeth as well as a permanent cosmetic treatment for discoloration. Following a comprehensive evaluation that included a thorough case history as well as extraoral, intraoral, clinical, and radiographic exams, discolorations and prior composite restorations were discovered. The vitality pulp test indicated the need for elective root canal therapy The patient was informed of the different treatment options. With the patient's permission, a comprehensive treatment plan was initiated following root canal therapy, which comprised zirconia crowns placed on the maxillary anterior teeth. During intraoral inspections, multiple composite restorations were detected. A porcelain fused metal crown was already present with 21 and the patient was not willing to undergo treatment with 21 for the zirconia crown. Maxillary and mandibular impressions were taken before scaling, polishing, and enhancing any existing restorations. Together with the dental technician, a diagnostic wax-up was created. The maxillary incisors were shaped using C1 shade composite resin. Apart from the root canal treatment, the teeth were prepared and posts made of fiber-reinforced composite were installed into the roots. Composite resin was used to create the cores, and the finish line was positioned just below the gingiva. A retraction cord was placed in the buccal gingival sulcus during impression recording, as seen in Figure 2, and full-arch impressions were created utilizing the putty reline technique and polyvinyl siloxane. After that, the impressions were filled in the dental laboratory, where Figure 3 illustrates the CAD-CAM fully stabilized zirconia coping procedure. Dentistry using emax (Ivoclar Vivadent) zirconia copings were layered carefully during the precise fabrication of Dentsply Sirona. A porcelain try-in, interocclusal adjustment, and confirmation were part of the final visit to ensure canine guidance and movements before glazing. Then, as seen in Figure 4, using resin cement zirconia crowns were cemented. Everything was done according to the manufacturer's specifications, including production, building, and cementation.

## Discussion

CAD/CAM zirconia ceramic prosthesis benefits include a wear pattern resembling tooth enamel, a tooth-like translucent look, and outstanding biological stability near the periodontium and oral tissues [16]. Lithium silicate ceramics are commonly used for multiple purposes, including veneers, tabletops, single crowns, and small anterior bridges. Moreover, we wish to incorporate bigger and more extensive restorations in the posterior area and expand the indication options. Then, according to Lohbauer et al. (2018), this goal can only be accomplished with CAD/CAM zirconia ceramic restorative materials [17]. The highly variable indication range of the third generation, which is the most commonly utilized, and the new fifth generation mixed zirconias within a CAD milling block are largely puzzling. The recommended treatment range for this generation ranges from the authorization of 14-unit bridges to the relatively small three-span bridges in the anterior region. As a result, the idea of a single zirconia material no longer being relevant; instead, a variety of material variants can be manufactured for customized uses [18].

To improve aesthetics, high-strength oxide ceramics were added as the core material; nonetheless, reports of layer chipping have surfaced. According to a study, after three years, bi-layered zirconia chipped at a rate of 24% as opposed to 34% for porcelain fused to metal. The high-strength monolithic zirconia with glazed and stained crowns have been assessed from numerous angles in recent years due to reports of chipping and fractures in the veneering ceramic. There is no additional ceramic veneering or layering on monolithic zirconia restorations that could chip or break. However, because this kind of ceramic is monochromatic and may be very opaque, it is thought to have less aesthetic value than traditional veneered crowns [19]. 

Zirconia-based prostheses have been demonstrated in clinical trials to be effective in long-term restorations [20]. However, there have been technical issues with zirconia-fixed prosthetic teeth and crowns that have been linked to their clinical efficacy. One such issue is the chipping and loss of veneered porcelain retention in zirconia framework systems [20]. The issues with chipping in veneering porcelain have not been resolved by machining the frames and veneers and stressing them with either a luting chemical or a fusing firing with CAD on. An alternate approach to lessening the problems associated with veneer chipping is the use of zirconia produced as a fully anatomical monolithic-shaped prosthesis. These are commonly referred to as monolithic zirconia crowns in prosthodontics [21]. Zirconia-toughened ceramics and alumina-toughened zirconia are two other varieties of zirconia. Additionally, as experimentally innovative zirconia forms with increased translucency, graded zirconia and nanostructured zirconia have been produced [22]. Zirconia toughened alumina is a comparatively recent variety of typical tough ceramics that is widely employed, primarily in oxide ceramic structural composites [23]. The microstructure of the material determines its mechanical properties, which are readily manipulated by densification and powder preparation procedures.

For our patient, the clinical results included the discoloration being covered up with long-lasting, biocompatible crowns. Additionally, the color matching led to increased self-worth and better social communication. Additionally, there was harmony in the smile line and the level of arrangement when speaking and smiling.

## Conclusions

Given the patient's emphasis on aesthetics, an all-ceramic restoration, more precisely, a multilayer zirconia restoration was the preferred course of therapy. Prosthetic teeth made of CAD/CAM zirconia have good biocompatibility, cause less wear on neighboring teeth, and offer stable color and long-lasting aesthetics. Restorations utilizing zirconia exhibit exceptional mechanical, chemical, and clinical performance, making them a promising prosthodontic substitute material. To sum up, using zirconia restorations in the maxillary anterior region offers a potential way to accomplish both functional and cosmetic goals. We have illustrated the benefits of zirconia through our case study, such as its biocompatibility, durability, and ability to resemble the esthetics of natural teeth. Zirconia crowns have been successfully used to restore the patient's smile, demonstrating its potential as an effective option for treating cosmetic issues in the anterior region. Nonetheless, further extensive clinical research is necessary to assess the efficacy and lifespan of zirconia restorations in this particular setting. However, our results show that zirconia is a useful material for getting the best results in anterior dental restorations, providing patients and dentists with a dependable way to improve smile aesthetics and restore function.
